# Clinical Efficacy and Safety of Teriparatide Versus Alendronate in Postmenopausal Osteoporosis: A Systematic Review of Randomized Controlled Trials

**DOI:** 10.7759/cureus.73068

**Published:** 2024-11-05

**Authors:** Sarvesh Nunkoo, Mooroogiah Krissheeven, Anusha Chitravanshi, Maheshwara Ramanah, Jared Robinson, Indrajit Banerjee

**Affiliations:** 1 Pharmacology, Sir Seewoosagur Ramgoolam Medical College, Belle Rive, MUS; 2 Surgery, Sir Seewoosagur Ramgoolam Medical College, Belle Rive, MUS

**Keywords:** alendronate, diphosphonates, osteoporosis, postmenopausal, teriparatide, treatment outcome

## Abstract

Alendronate, a second-generation bisphosphonate, remains the first-line therapeutic option for postmenopausal osteoporosis. It acts on the bone resorbing osteoclasts causing their apoptosis. This is achieved by producing toxic adenosine triphosphate (ATP) analogues and interfering with the mevalonate pathway. Teriparatide, a recombinant form of parathyroid hormone, is an alternative option to this more conventional drug. It is an anabolic drug that mediates its biological effect via specific, high-affinity membrane cell-surface receptors expressed on the osteoblasts. It promotes bone formation more than bone resorption. Hence, this research was conducted to delineate the effectiveness and clinical safety of teriparatide as compared to alendronate in women suffering from postmenopausal osteoporosis. An extensive search was conducted through PubMed, Google Scholar, Trip (Turning Research into Practice), and Cochrane Central Register of Controlled Trials (CENTRAL) including studies published between August 2017 and October 2024 in accordance with Preferred Reporting Items for Systematic Reviews and Meta-Analyses (PRISMA) guidelines 2020. The Medical Subject Headings (MeSH) terms and Boolean operators used were "Alendronate" OR “Diphosphonates” OR "Teriparatide" OR “Treatment Outcome” OR “Postmenopause” AND "Osteoporosis". Randomized controlled trials were included in this systematic review. Five full-text articles were ultimately considered and critical appraisal was performed thereon. The annual incidence rate of morphometric vertebral fracture in the sequential therapy (teriparatide) group (0.1020 and 0.1334) was significantly lower than monotherapy (alendronate) (0.1492 and 0.1734). Quality of life (QoL) by week 12 was better in teriparatide than alendronate and no patient encountered any severe adverse effects with teriparatide after 72 weeks of treatment. Thus, based on the results, teriparatide is more effective than alendronate in increasing the bone mineral density (BMD) of L2-4 vertebrae and the hip bone. However, alendronate is better in the case of femoral neck fractures. Furthermore, spinal strength shows a better response in the trabecular than peripheral compartment with teriparatide. Teriparatide is also safer than alendronate due to its lower incidence rate in morphometric vertebral fracture, lack of severe adverse effects and better QoL. Teriparatide showed comparable inhibition of vertebral collapse, increase in BMD, promotion of bone union, and improvement of pain.

## Introduction and background

Osteoporosis is a systemic bone disease that creates an imbalance in bone remodelling. This is primarily due to failure to achieve bone peak mass and excessive bone resorption. As such, there is decreased bone strength and increased susceptibility to bone fractures. Some clinically important risk factors include age, sex, body weight, a history of prior fractures, smoking, glucocorticoid (steroid) therapy, and rheumatoid arthritis. Diagnosis is based on the measurement of bone mineral density using a dual X-ray absorptiometry (DXA) considered the gold standard [1]. Menopause and osteoporosis are interconnected. Menopause signifies the final menstrual cycle of women. It occurs between the ages of 45 and 55 years. The lowered oestrogen levels which occur in postmenopausal women result in a higher chance of developing osteoporosis [2]. The World Health Organization (WHO) states that osteoporosis affects 21.2% of women over the age of 50 years and that up to 70 fragility fractures occur every minute in those aged above 55 years globally [3,4]. However, male osteoporosis is also an alarming health problem. One in three osteoporotic fractures occur in men and fracture-related mortality and morbidity are even higher than in women. Interestingly, oestrogen, and not testosterone, appears to play a role in regulating male skeletal status [5]. 

Medications to treat osteoporosis can be categorized as either antiresorptive (i.e., bisphosphonates, oestrogens, calcitonin, and denosumab) or anabolic (i.e., teriparatide and abaloparatide). The antiresorptive medications mainly decrease bone resorption whereas the anabolic ones increase bone formation more than bone resorption [6]. Among these, bisphosphonates and parathyroid hormones are mostly preferred to prevent bone fractures and enhance bone proliferation and stability which will ultimately reduce the osteoporosis risk [7]. Bisphosphonates remain the first-line therapeutic option for postmenopausal osteoporosis [6]. They act on the bone-resorbing osteoclasts by causing their apoptosis. This is achieved by producing toxic ATP analogues and interfering with the mevalonate pathway [8]. Some studies show that nitrogen-containing-bisphosphonates like alendronate and risedronate lower the incidence of both vertebral and non-vertebral fractures by approximately 50%. Other bisphosphonates such as etidronate and ibandronate are mostly effective in the locality of the vertebral column [9]. Alendronate, 10 milligrams (mg) once a day or 70 mg given once weekly produces a greater increase in BMD relative to risedronic acid given once weekly at the lumbar spine. Clinicians therefore most often prefer to select alendronate over the other bisphosphonates as a treatment option [10]. 

Teriparatide, a recombinant form of human parathyroid hormone, has also proved its efficacy and safety in the treatment of osteoporosis. It consists of the first 34 amino acids of the parathyroid hormone, which is the bioactive portion responsible for its effects on bone metabolism. It is usually given subcutaneously at a dose of 20 micrograms (μg)/day for a period of 11-21 months. Teriparatide mediates its biological effect via specific, high-affinity membrane cell-surface receptors expressed on the osteoblasts. Due to its anabolic effect, the drug promotes bone formation more than bone resorption and thereby combats osteoporosis. Teriparatide (Forteo) has shown promising effects on bone structure, strength and quality. Despite its duration of treatment and high cost, teriparatide is well tolerated by many patients and it shows a high rate of compliance [11].

Even with its wide popularity, alendronate has some limitations such as oesophageal problems, hypocalcemia, and osteonecrosis of the jaw. It is also not recommended if the patient cannot stand upright or stand for at least 30 minutes. Likewise, the concern of osteosarcoma with teriparatide is still there. No consensus has been made to date on the best medication for these patients. Many randomized controlled trials (RCTs) have been conducted on patients of postmenopausal osteoporosis receiving teriparatide and alendronate either as a sequential therapy or monotherapy during the last few decades. This systematic review has been conducted by comparing the clinical efficacy and safety of teriparatide to alendronate in postmenopausal osteoporosis.

This systematic review of RCTs aims to evaluate the changes in bone mineral density of total hip, femur, and lumbar vertebrae, the incidence of morphometric vertebral fractures, and both the safety and adverse effects encountered after treatment with teriparatide and alendronate.

## Review

Methodology

The Preferred Reporting Items for Systematic Reviews and Meta-Analysis (PRISMA) 2020 guidelines were followed.

Literature Search

A thorough search was done on PubMed, Trip (Turning Research into Practice), Google Scholar, and Cochrane databases in order to identify relevant manuscripts. A combination of keywords and boolean operators was used for data extraction (((((Alendronate) OR (Diphosphonates)) OR (Teriparatide)) OR (Treatment Outcome)) OR (Postmenopause)) AND (Osteoporosis). The strategy used in the search as well as the total number of articles screened are shown in Table 1.

**Table 1:** Various databases searched, boolean operators and MeSH terms used MeSH: Medical Subject Headings; Trip: Turning Research into Practice

Databases searched	Boolean operators and keywords	Total number of articles
PubMed	(((((Alendronate) OR (Diphosphonates)) OR (Teriparatide)) OR (Treatment Outcome)) OR (Postmenopause)) AND (Osteoporosis) Filters: English, from 2017 - 2024	9622
Cochrane Central Register of Controlled Trials (CENTRAL)	(((((Alendronate) OR (Diphosphonates)) OR (Teriparatide)) OR (Treatment Outcome)) OR (Postmenopause)) AND (Osteoporosis) in All Text Filters: English, from 01.08. 2017 - 15.10. 2024	8358
Trip	(((((alendronate) OR (diphosphonates)) OR (teriparatide)) OR (treatment outcome)) OR (postmenopause)) AND (osteoporosis) Filters: from_date:01.08.2017 to_date:15.10.2024	4136
Google Scholar	(((((alendronate) OR (diphosphonates)) OR (teriparatide)) OR (treatment outcome)) OR (postmenopause)) AND (osteoporosis) filters: Custom range- 2017-2024	23900
		46, 016

Inclusion and Exclusion Criteria

Inclusion criteria: All RCTs providing information on teriparatide and alendronate relevant to post-menopause osteoporosis, between August 1, 2017, and October 15, 2024, were assessed and were included in the study. All the articles were screened independently by four researchers (SN, MK, AC, and IB) in all fields. In case of discrepancy, the fifth researcher (JR) was consulted. Full-text articles were included and accessed for eligibility in this systematic review. All the RCTs available in English literature were included in the study.

Exclusion criteria: Data resources such as non-RCTs, cohort studies, case-control studies, cross-sectional studies, case series, case reports, in vitro studies, and animal experiments were disregarded. All commentaries, letters to the editor, expert opinion, and review articles were omitted from this systematic review.

Methodology Quality Assessment

The quality assessment of the selected RCTs was assessed independently by four researchers (SN, MK, AC, and MR). The Cochrane risk-of-bias tool for randomized trials (RoB2) was used for quality assessment. The RoB2 tool is best suited and implemented to assess the domains at low, unclear, and high risk of bias. The Risk-of-bias Visualization (ROBVIS) tool is a web-based application program that was used to generate the traffic light plots and the weighted bar plots.

Extracted Data

The data from the final RCTs included in the analysis were synthesized by four independent researchers. The data was extracted in a tabular form based on authors, years of study, study design, sample size and age group of patients, BMI, follow-up, dose, duration of adverse drug reaction, limitations, and outcome of therapy.

Results

**Figure 1:**
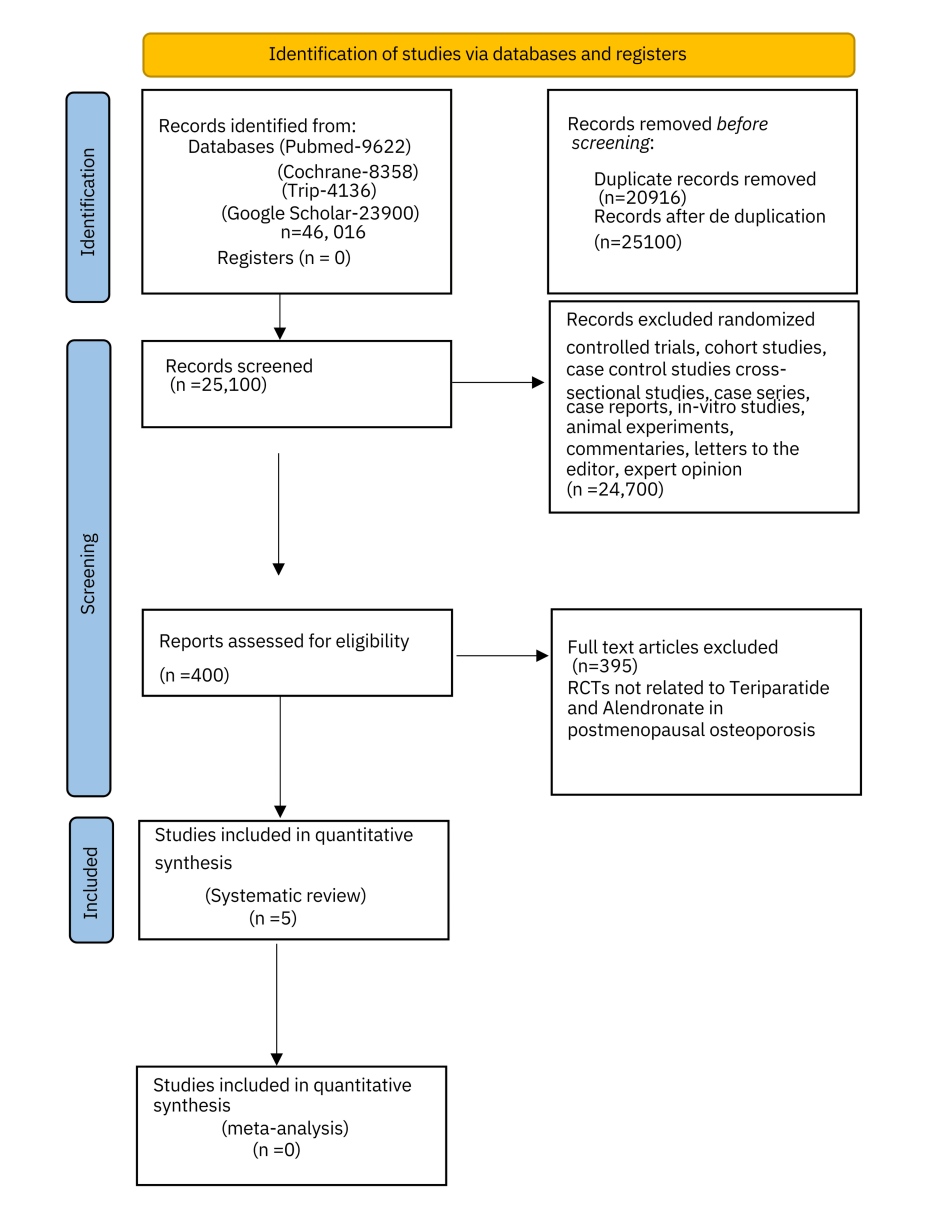
PRISMA 2020 flowchart PRISMA: Preferred Reporting Items for Systematic Reviews and Meta-Analysis

The literature search yielded a total of 46,016 articles (PubMed: 9622, Cochrane: 8358, Trip: 4136, and Google Scholar: 23900). Among these, 20,916 were noted as duplicates and were excluded from the initial analysis. Thus, 25,100 manuscripts were screened after deduplication. Non-RCTs, cohort studies, cross-sectional studies, case-control studies, case series, case reports, in vitro studies, animal experiments, commentaries, letters to the editor, and expert opinions were additionally excluded (n=24,700). A total of 400 full-text articles were assessed for eligibility. An in-depth evaluation and analysis further excluded 395 from the analysis quality assessment as these were not related to teriparatide and alendronate in postmenopausal osteoporosis. Five RCTs were finally assessed regarding the efficacy and safety of teriparatide vs alendronate in postmenopausal women suffering from osteoporosis and were hence included in the systematic review for qualitative synthesis (Figure 1).

Risk of Bias Analysis

**Figure 2:**
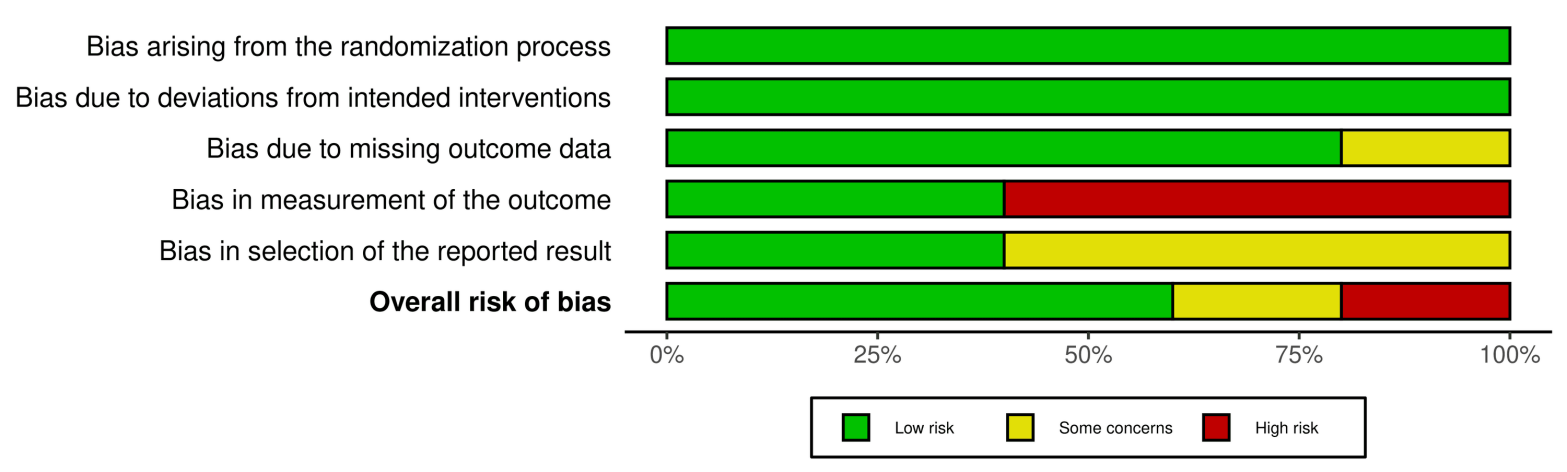
Weighted bar plots showing the summary of risk of bias for the RCTs based on five domains RCTs: randomized controlled trials

**Figure 3:**
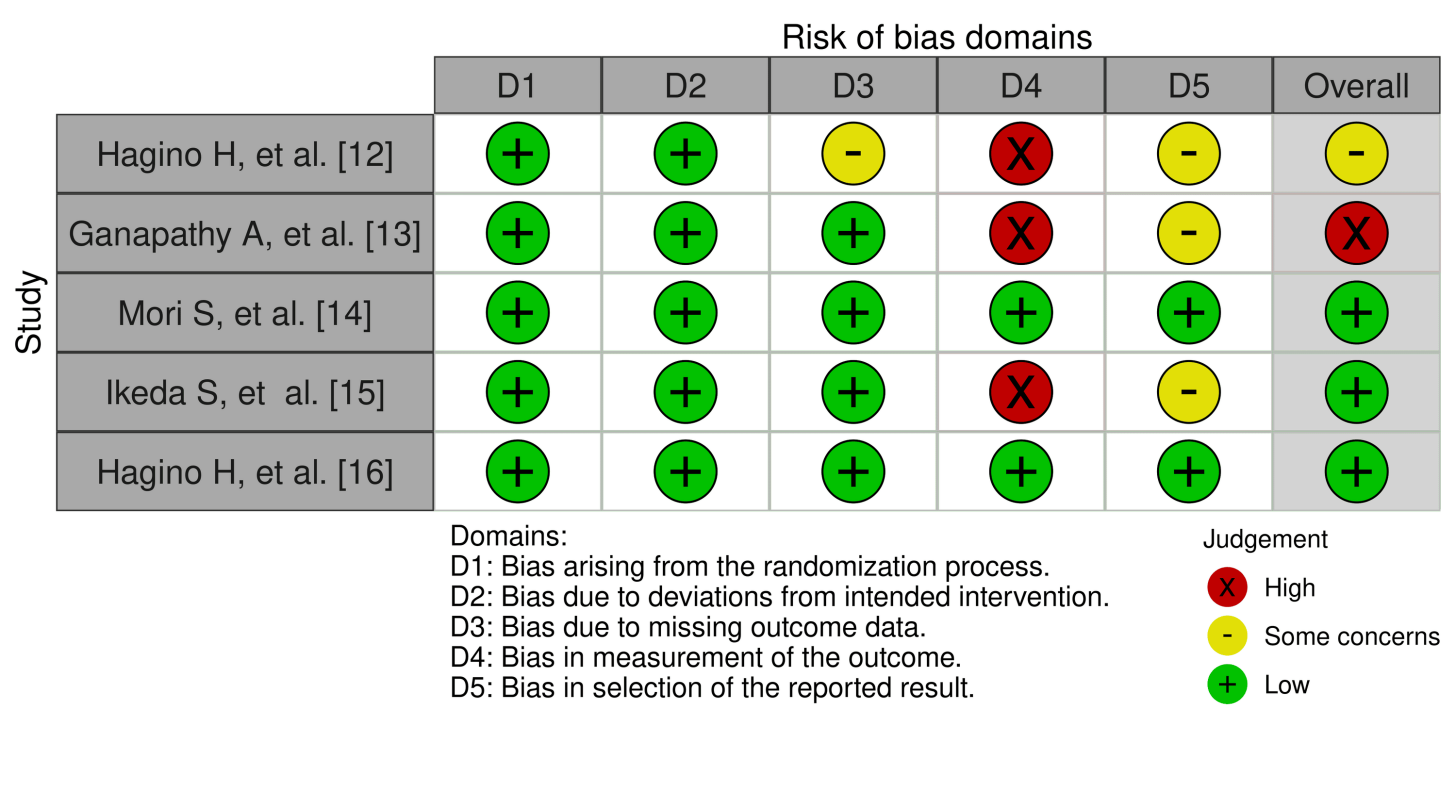
Traffic light plot showing the risk of bias of the five RCTs included in the final review RCTs: randomized controlled trials. Reference: [12-16]

All the included RCTs underwent a quality assessment by the ROB2 tool which showed good overall results of low risk of bias in the randomization process(100% low risk), deviations from intended interventions (100% low risk), missing outcome data (low risk 80%), measurement of outcome (low risk 40%, high risk 60%) and selection of reported result (low risk 40%, some concerns 60%), and overall risk of bias for the five RCTs were found to be low risk 60%, some concern 20%, and high risk 20%, which signified some concerns (Figures 2, 3).

Table 2 depicts the author, year of publication, study design, sample size, and age group of patients. Table 3 depicts author, year, BMI, follow-up period, dose, duration of ADR, limitations, and outcome of therapy.

**Table 2:** Study design, sample size, interventional group, control, and age group TPTD: teriparatide; ALN: alendronate; BMD: bone mineral density; vBMD: volumetric bone mineral density; QoL: quality of life

Author, year	Study design	Sample size	Interventional group	Control	Patient age
Hagino et al., 2024 [12]	RCT	559	202	357	Women aged 75 years or older; mean age = 81 years
Ganapathy et al., 2023 [13]	RCT	86	28	58	Women aged 54-70 years
Mori et al., 2023 [14]	RCT	1011	505	506	Women aged at least 75 years
Ikeda et al., 2019 [15]	RCT	96	48	48	Women aged at least 65 years
Hagino et al., 2021 [16]	RCT	1011	505	506	Women aged at least 75 years

**Table 3:** BMI, follow-up period, dose, duration of treatment, ADR, limitations, and outcome of therapy - indicates no statistical improvement in patient outcomes after treatment; + indicates statistical improvement in patient outcome after treatment. TPTD: teriparatide; ALN: alendronate; BMD: Bone mineral density; vBMD: volumetric bone mineral density; QoL:quality of life; qw: once weekly; DXA: dual energy X-ray absorptiometry; ADR: adverse drug reaction; QCT: quantitative computed tomography

Author, year	BMI (kg/m^2^)	Follow-up period	Dose	Duration of treatment	ADR developed during treatment	Potential limitations	Outcome of therapy (+/-)
Hagino et al., 2024 [12]	TPTD-ALN Group: 22.3±3.7; ALN Group: 22.3±3.4	BMD was measured at 24 weeks, 48 weeks, 72 weeks, and 120 weeks for both groups by DXA	TPTD 56.5 μg was injected qw; ALN: Tab 5 mg OD or Tab 35 mg qw or 35 mg jelly or 900 μg infusion once every four weeks	1.5 years	No ADR reported	This study included both women with high risk of bone fracture and those with a low risk of bone fracture	+
Ganapathy et al., 2023 [13]	Daily Group: 23.7±2.99; Cyclic Group: 24.6±5.47	QCT scans were taken at two years and four years	Daily group: TPTD 20 μg daily; Cyclic group: 20 μg daily for three months followed by months off	4 years	No ADR reported	Small sample size	+ (- in hip only)
Mori et al., 2023 [14]	Sequential therapy: 22.2±3.8; Monotherapy: 22.1±3.5	BMD was measured at 0, 12, 24, 48, 72, 120 weeks by DXA	TPTD 56.6μg; ALN: Tab 5mg orally OD or 35mg tablet or jelly orally qw or 900μg infusion bag i.v every 4 weeks	120 weeks	Infections and infestations, GI disorders, musculoskeletal and connective tissues disorders (more in sequential therapy)	Small sample size and The second part treatment of ALN followed by TPTD was only 48 weeks	+
Ikeda et al., 2019 [15]	TPTD: 21.3; ALN: 21.1	X-ray images/DXA were taken at weeks 1, 2, 4, 8, and 12	Injection of TPTD 56μg qw ALN:35mg per week	12 weeks	No ADR reported. Significant decrease in pain and better QoL in TPTD group	Small sample size	+
Hagino et al.; 2021 [16]	TPTD: 22.2±3.8; ALN: 22.1±3.5	BMD was measured at 0, 12, 24, 48, 72, 120 weeks by DXA	PTD 56.6 μg ; ALN: Tab 5 mg orally OD or 35 mg tablet or jelly orally qw or 900 μg infusion bag IV every four weeks	120 weeks	Mild to moderate ADR three deaths (two in TPTD group and one in ALN group) occurred and could be possibly related to the treatment	Small sample size	+

Discussion

The benefits of taking teriparatide are likely to outweigh any possible risks in postmenopausal women suffering from osteoporosis. Teriparatide acts as an anabolic agent and is reported to reduce the risk of fracture by increasing bone formation, osteoblast differentiation, osteoblast function, and survival. Alendronate has the potential ability to inhibit osteoclastic bone resorption.

This systematic review assessed the progressive impact of teriparatide and alendronate on bone remodelling as well as bone resorption. Serial BMD testing therapy is done to assess the lumbar spine, hip, and proximal femur as a standard means of identifying patients with osteoporosis. We speculated that teriparatide treatment for a longer period of time will not only be effective in increasing the lumbar and hip BMD but also in reducing the risk of vertebral and non-vertebral fractures. Ultimately, in sequential therapy, teriparatide is superior to alendronate due to its lower incidence rate in morphometric vertebral fractures. In this study, evidence of teriparatide with patients suffering from osteoporosis at a high risk of fracture was shown to have favourable effectiveness and safety profiles as opposed to alendronate. 

Hagino et al. (2024) conducted an RCT among 559 Japanese women with primary osteoporosis, of which 202 intervention patients received sequential therapy of teriparatide-alendronate and 357 control patients received monotherapy of alendronate for a period of 120 weeks by dual-energy X-ray absorptiometry [16]. BMD ≥ -2.5 SD at 72 weeks in the L2-4, total hip, and femoral neck sites was 13.6%, 15.2%, and 5.3%, respectively, in the teriparatide-alendronate group and 15.6%, 9.6%, 7.1%, respectively, in the alendronate group. The achievement rate in the total hip BMD was higher in the teriparatide-alendronate group (15.2%) than in the alendronate group (9.6%). However, it was not statistically significant and it should be verified by studies with appropriate sample size [11]. A similar study conducted by Chiba et al. substantiates the fact that after 18 months of treatment the BMD of total hip increased by +3%, with daily 20 μg of teriparatide (D-PTH), by +2% with weekly 56.5 μg of high-dose teriparatide (W-PTH) and by 3% with bisphosphonates (BP) which include alendronate 35 mg/week or risedronate 17.5 mg/week [17].

Ganapathy et al compared the effects of a four-year cyclic regimen versus a two-year daily regimen of teriparatide in postmenopausal women with osteoporosis [13]. Quantitative computed tomography (QCT) images were acquired at baseline and induced a comprehensive alteration in peripheral and trabecular compartments from daily and cyclic teriparatide administration. Nevertheless, the bone strength at the hip and the spine, volumetric integral, trabecular, and cortical bone mineral density (vBMD) were analyzed. The spine strength increased by up to 14% in daily treatment and up to 16% in cyclic therapy (p<0.001). Similarly, the daily group had an increased total hip density (3%, p<0.05) and total hip strength (5%) with additional gains in hip BMD with alendronate treatment from two to four years. The cyclic group increased only for trabecular hip density (6%, p<0.001). However, there was no significant group difference between any of these variables. In the study conducted by Takahashi et al., a QCT was done which concluded that D-PTH after 18 months had a strong effect on the trabecular bone of vertebra and no significant increase in the cortical vBMD, as compared to W-PTH and BP, which increased the cortical BMD and BV of the proximal femur [18]. D-PTH is more efficacious on the lumbar spine than with BP (D-PTH +10.3%, BP +5.5%). The limitations of their study were the resolution of the clinical CT and a small sample size.

Hagino et al.'s 2021 RCT evaluated the incidence of morphometric vertebral fractures in patients receiving teriparatide (n=251) and alendronate (n=357) [16]. From the results, it can be inferred that the teriparatide group had a much lower incidence rate (0.1334) than the alendronate group (0.1734). The study's main limitation was its small sample size. A similar study by Mori et al. proved that the incidence of morphometric vertebral fracture during 120 weeks in the sequential (teriparatide) group had a lower incidence rate (0.1020) than that of the alendronate group (0.1492) [14].

In a study conducted by Ikeda et al., QoL was evaluated in 96 postmenopausal women with new-onset osteoporotic vertebral fractures [15]. Participants were divided into two groups of 48, receiving either teriparatide or alendronate. Their findings showed that weekly teriparatide significantly improved the QoL, reduced vertebral collapse, increased BMD, promoted bone union, and reduced pain in patients [15].

A long‐term toxicology study conducted by Krege et al. in rats showed evidence of osteosarcoma after near-lifetime exposure to teriparatide with 19 months of treatment [19]. The clinical trial data from this study was initially and subsequently evaluated, and results showed reductions in vertebral fractures, nonvertebral fractures, and back pain, and increased BMD.

There are several studies investigating the sequential strategy. Lindsay et al. showed that adding teriparatide to an ongoing hormone replacement therapy in postmenopausal women resulted in a superior gain in BMD compared with women continuing oestrogen supplementation only [20].

Cosman et al. compared the effect of switching versus adding teriparatide in postmenopausal women on treatment with either raloxifene or alendronate for at least 18 months [21]. The biochemical bone turnover response to teriparatide was reduced in add versus switch patients. Concomitantly, the bone density responses were greater in add versus switch patients in both the spine and hip in the alendronate group and in the hip in the raloxifene group.

Chandran conducted a study where, after 18-36 months of treatment with alendronate or raloxifene, a switch to teriparatide was made [22]. A decrease in hip BMD at six months was found in those previously treated with alendronate but not those who had received raloxifene. Moreover, cyclic teriparatide over two years is shown to improve BMD similarly to daily treatment in women who are on ongoing alendronate therapy, despite receiving only 50% of the usual teriparatide dose. Hence, sequential therapy with an antiresorptive drug following teriparatide increases bone mass and supports the secondary mineralization of newly formed bone.

Panico et al. showed that the most commonly reported adverse effects seen in the first month of treatment with teriparatide were back pain, headache, dizziness, nausea, and vomiting. The most common adverse effects associated with alendronate were abdominal pain, arthralgia, and dyspepsia; tolerability of alendronate was comparable to teriparatide [23]. Furthermore, Black et al. showed that bone resorption decreased in the combination therapy group and the alendronate group [24]. In their study, there was a significant increase in the mean serum uric acid concentration in both the parathyroid hormone group (61 μmol/L) and the combination-therapy group (51 μmol/litre); whereas there was no change in the alendronate group. Three women had gout, one in the parathyroid hormone group and two in the combination therapy group.

## Conclusions

Teriparatide should be used as the first-line treatment as it shows a greater improvement in the BMD of hip bone and L2-4 vertebrae along with the promotion of bone union and bone strength as opposed to alendronate. Teriparatide is considered to be superior to alendronate resulting in a lower incidence rate in morphometric vertebral fractures as opposed to monotherapy with alendronate in women suffering from postmenopausal osteoporosis. 
